# Analysis and pharmacological modulation of senescence in human epithelial stem cells

**DOI:** 10.1111/jcmm.17434

**Published:** 2022-06-15

**Authors:** Vanessa Barbaro, Antonio Orvieto, Gualtiero Alvisi, Marina Bertolin, Filippo Bonelli, Thomas Liehr, Tigran Harutyunyan, Stefanie Kankel, Gordana Joksic, Stefano Ferrari, Elena Daniele, Diego Ponzin, Daniela Bettio, Leonardo Salviati, Enzo Di Iorio

**Affiliations:** ^1^ Fondazione Banca degli Occhi del Veneto Venice Italy; ^2^ Department of Computer Science ETH Zurich Zurich Switzerland; ^3^ Department of Molecular Medicine University of Padua Padua Italy; ^4^ Jena University Hospital, Friedrich Schiller University Institute of Human Genetics Jena Germany; ^5^ Department of Genetics and Cytology Yerevan State University Yerevan Armenia; ^6^ Department of Physical Chemistry, Vinča Institue of Nuclear Sciences University of Belgrade Vinča Serbia; ^7^ Clinical Genetics Unit University Hospital of Padua Padua Italy; ^8^ Department of Women and Children’s Health University of Padua Padua Italy

**Keywords:** DAPT (γ‐secretase inhibitor; N‐[N‐(3,5‐difluorophenacetyl)‐L‐alanyl]‐S‐phenylglycine T‐butyl ester), EEC syndrome, lifespan, p63, RNAseq, self‐renewal, stem cells

## Abstract

Human epithelial stem cells (ESCs) are characterized by long‐term regenerative properties, much dependent on the tissue of origin and varying during their lifespan. We analysed such variables in cultures of ESCs isolated from the skin, conjunctiva, limbus and oral mucosa of healthy donors and patients affected by ectrodactyly‐ectodermal dysplasia‐clefting syndrome, a rare genetic disorder caused by mutations in the p63 gene. We cultured cells until exhaustion in the presence or in the absence of DAPT (γ‐secretase inhibitor; N‐[N‐(3, 5‐difluorophenacetyl)‐L‐alanyl]‐S‐phenylglycine T‐butyl ester). All cells were able to differentiate in vitro but exhibited variable self‐renewal potential. In particular, cells carrying p63 mutations stopped prematurely, compared with controls. Importantly, administration of DAPT significantly extended the replicative properties of all stem cells under examination. RNA sequencing analysis revealed that distinct sets of genes were up‐ or down‐regulated during their lifetime, thus allowing to identify druggable gene networks and off‐the‐shelf compounds potentially dealing with epithelial stem cell senescence. These data will expand our knowledge on the genetic bases of senescence and potentially pave the way to the pharmacological modulation of ageing in epithelial stem cells.

## 1 INTRODUCTION

**FIGURE 1:**
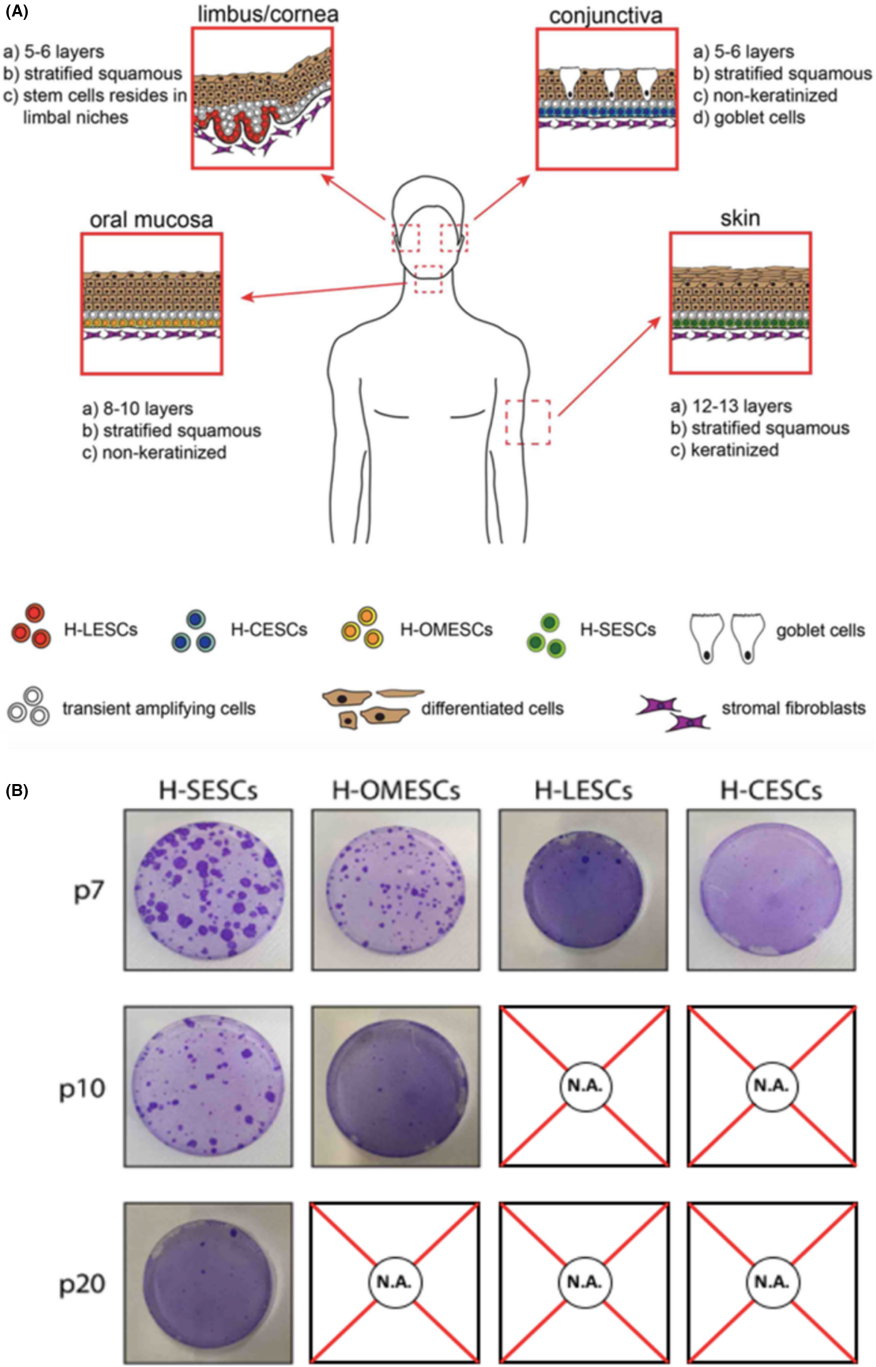
Comparative features of stem cells obtained from four different epithelial tissues. (A) Model of the four epithelia investigated in this study: skin, oral mucosa, cornea and conjunctiva and their organization at tissue level. (B) Colony forming efficiency of H‐SESCs, H‐OMESCs, H‐LESCs and H‐CESCs evaluated at the last passage in culture. H‐SESCs, human skin epithelia stem cells; H‐OMESCs, human oral mucosa epithelial stem cells; H‐LESCs, human limbal epithelia stem cells; H‐CESCs, human conjunctival epithelial stem cells

The homeostasis of the epithelial tissues is ensured by the presence of adult stem cells (SCs), which are endowed with two important features: self‐renewal and unlimited potency.^1^ Recent evidence has demonstrated that in vivo SCs reside in specific regions of the tissue and remain quiescent until activation is required either due to the normal need to maintain tissues or in response to diseases and injuries.^2^ The structure and localization of such niches are tissue‐specific and depend on tissue turnover and regenerative potential. The ability of SCs to survive and retain their proliferative potential throughout their lifespan does not necessarily imply that they have an endless capacity to divide, undergoing constant self‐renewal. As tissues have different developmental needs and cellular turnover rates, the in vivo self‐renewal frequencies of SCs are different. Interestingly, the behaviour of the different epithelia reflects the physiological role of the tissues they belong to. The epidermis undergoes keratinization, a process in which the epidermal cells progressively mature from basal cells with proliferative potential to the lifeless, flattened squames of the *stratum corneum* to generate a functional barrier to the external environment. The incessant exposition to thermal shocks, pathogens and chemical agents prompts the skin epithelium to undergo a continuous replacement of cells, which involves cell proliferation and differentiation that culminate in the desquamations of keratinized flakes. The homeostasis is guaranteed by epithelial stem cells (ESCs) located in the interfollicular epidermis (IFE), with a turnover time of 28 days.^3^ The oral mucosa, that protects the oral cavity from bacteria and from the mechanical stress during mastication, has similar functions. It is possible to distinguish the different areas of the mucosa according to differences in keratinization. The gingivae and hard palate are keratinized, while the floor of the mouth, the buccal regions located under the surface of the tongue, and the sulci are not keratinized. The oral mucosa has higher numbers of cell layers compared with the epidermis (Figure 1A).^4^, ^5^, ^6^ Quiescent cells are located in the basal layer, while proliferation is restricted to the parabasal and adjacent suprabasal layer.^6^, ^7^, ^8^, ^9^, ^10^, ^11^, ^12^

The ocular surface, which is composed of the cornea centrally and the sclera peripherally, shares the same ectodermal origin. Located in the outermost part of the eye, the cornea is vulnerable to damages caused by burns, abrasions, contact lenses, alterations in tear production, infections and other diseases.^13^ The corneal epithelium consists of a single basal layer and of stratified squamous epithelial cells and is essential to guarantee the structural uniformity and the transparency of the tissue. During corneal homeostasis, human limbal epithelial stem cells (H‐LESCs) proliferate and generate transient amplifying cells (TA) that divide, differentiate and migrate to the centre of the cornea to regenerate the epithelium.^13^ H‐LESCs are present in the basal layer of the limbus, in specific niches called Palisades of Vogt.^14^ Unlike the H‐LESCs, the precise location of the human conjunctival stem cells (H‐CESCs) is still controversial. Although previous studies suggested that H‐CESCs are uniformly distributed throughout the whole ocular surface,^15^, ^16^ recent reports have demonstrated that they are located in the fornix and/or in the bulbar conjunctiva.^17^, ^18^ In fact, the fornix may provide greater physical protection, intraepithelial mucous crypts, vasculature and immune cells, typical features of the SC niches.

If SCs are exhausted too quickly, or if genetic defects alter their proliferative potential, tissue atrophy and premature ageing can arise. For example, mutations in the *p63* gene, known to cause ectrodactyly‐ectodermal dysplasia‐clefting (EEC) syndrome, induce a rapid exhaustion of clonogenic and self‐renewal potential of ESCs, resulting in accelerated ageing.^19^, ^20^, ^21^ Conversely, mutations that promote frequent SC divisions without an appropriate differentiation balance can result in abnormal tissue development and even cancer.^22^

In this study, we demonstrate that the frequency and timing of SC divisions are tightly regulated and specific for each tissue, as well as preserved in vitro to ensure a defined lifelong maintenance of the SCs population. In addition, the treatment of cells with the γ‐secretase inhibitor DAPT (N‐[N‐(3,5‐difluorophenacetyl)‐L‐alanyl]‐S‐phenylglycine T‐butyl ester) resulted in the extension of the lifespan of ESCs. Finally, we identified several genes strongly related to the senescence process, by means of RNA sequencing (RNAseq) and gene ontology (GO) analyses, reconstructed the protein networks involved and identified potential ageing modulating drugs.

## 2 MATERIALS AND METHODS

### 2.1 Ethics declarations

Samples were obtained from donors and patients with informed written consent after approval from the Ethical Committee for Clinical Trials of Padua, Italy (Prot. 4503/AO/18). Human corneas unsuitable for transplantation and duly consented for research by the donor's next of kin were obtained by Fondazione Banca degli Occhi del Veneto (Venice, Italy). The forms to obtain consent for harvesting corneal tissues followed the standards set by the Italian National Transplant Service (Centro Nazionale Trapianti – Rome, Italy). The research was performed in accordance with the Declaration of Helsinki.

### 2.2 Cell culture

Primary human keratinocytes were isolated from fresh skin, conjunctiva, oral mucosa and limbus biopsies (*N* = 12) of healthy donors, aged between 40 and 50 years, and from oral mucosa biopsies (*N* = 6) of three patients with EEC syndrome. Once isolated, cells were cultivated and serially propagated, as previously described.^20^ To calculate cell doublings and cumulative population doublings the formula we used was 3322*log10 (UCY/I), where UCY = the cell yield at that passage and I = number of clonogenic cells. The clonogenic cells were calculated as the number of cells used as inoculum to begin a subculture multiplied by the colony forming efficiency (CFE) value.

Clonal analysis was performed to obtain holoclones, meroclones and paraclones, as previously described.^23^ DAPT was administered as previously described.^24^

### 2.3 Evaluation of the relative telomere length

Cytogenetic analysis of cells was performed according to standard procedures.^25^ Fluorescence in situ hybridization (FISH) was done on chromosome preparations according to manufacturer's recommendations and as previously reported.^26^ Commercially available all‐telomere probes were applied (Telomere PNA Kit, Agilent, Santa Clara, CA, USA). Pictures were taken with a Zeiss Axioplan Imaging microscope (Jena, Germany and ISIS software (MetaSystems, Altlussheim, Germany).

Telomere FISH signals were quantified on a per‐cell basis using the open‐source plugin “Telometer” program (version 3.0.6, available at: https://demarzolab.pathology.jhmi.edu/telometer/). FISH images were converted to 16‐bit grayscale TIFF images using the ImageJ program (available at: https://imagej.nih.gov/ij/). The images with telomeric signals were normalized by background subtraction via comparison with matching images of DAPI (4′, 6‐diamidino‐2‐phenylindole). A region of interest (ROI) with telomeric signals was selected in pictures of DAPI‐stained cells using the freehand selection tool in the ImageJ program. Telomeric signals within each ROI were then measured with the ImageJ program and the data of fluorescence intensity and area for each telomeric signal were obtained. RTL of each cell was calculated by multiplying the fluorescence intensity value of each telomeric signal with their corresponding areas and shown as arbitrary units.^27^ Single values of RTL for each cell were obtained and the comparison of 10–20 RTL values was performed using Statgraphics Centurion (version 16.2) statistical program. Normal distribution of data was evaluated. Differences between means were determined through a Multiple Range Test using Bonferroni's analysis. Statistical significance was set at *p* < .05.

### 2.4 Mitochondrial activity assay

MitoTracker®Red CMXRos (Invitrogen, M7512) staining was performed following manufacturer's instructions. Briefly, cells were trypsinized and centrifuged on tissue slides. Mitotracker was dissolved in DMEM (Dulbecco's Modified Eagle's Medium) at a final concentration of 250 nM and cells incubated for 45 min at 37°C. Cells were fixed with 4% PFA (paraformaldehyde; ChemCruz, sc‐281,692) and permeabilized with 1x TrytonTM (Sigma‐Aldrich, T8787). Slides were mounted using DAPI Fluoromount‐GTM (Electron Microscopy Sciences, CAT #17984–24). Pictures were captured using the camera incorporated in Nikon Eclipse Ti microscope. Corrected total cell fluorescence (CTCF) was finally calculated by using ImageJ software (National Institutes of Health, Bethesda, MD, USA).

### 2.5 RNA isolation, library preparation and sequencing

Total RNA was isolated from cultured keratinocytes at each passage and subsequently purified with the RNeasy Clean‐Up Kit (Qiagen, Valencia, CA, USA). TruSeq Stranded mRNA Sample Prep kit (Illumina, San Diego, CA) was used for the library preparation following the manufacturer's instructions, starting with 1–2 μg of RNA. The poly‐A mRNA was fragmented for 3 min at 94°C and purification performed by using 1X Agencourt AMPure XP beads. Both RNA samples and final libraries were quantified by using the Qubit 2.0 Fluorometer (Invitrogen, Carlsbad, CA) and quality tested by Agilent 2100 Bioanalyzer RNA Nano assay (Agilent technologies, Santa Clara, CA). Libraries were then processed with Illumina cBot for cluster generation on the flowcell, following the manufacturer's instructions and sequenced on single‐end (or paired‐end, if required) mode at the multiplexing level requested on HiSeq2500 (Illumina, San Diego, CA). The CASAVA 1.8.2 version of the Illumina pipeline was used to process raw data for both format conversion and de‐multiplexing.

### 2.6 RNAseq data elaboration

The data set contains Fragments Per Kilobase Of Exon Per Million Fragments (FPKM) estimates (using Cufflinks) for 27,028 genes and 49,772 isoforms. Data comes from four different cell cultureswhich we will refer to as LH, LH‐DAPT, OMESCs and EEC‐OMESCs. For each cell culture, cDNA (for LH and DAPT‐LH) or RNA (for OMESCs and EEC‐OMESCs) was collected and analysed using a TruSeq Stranded mRNA Sample Prep kit (Illumina, San Diego, CA) at three different cell passages in culture (as already described).

We performed the following preprocessing steps (in order):

1. 21 genes and 62 isoforms were removed due to measurement errors (FPKM status: fail);
2. 82.12% of genes and 91.51% of isoforms are not expressed in a significant way in at least one of the cell cultures (sum of FPKMs across the three time‐points less than 5, or initial FPKM less than 1). We did not consider these genes for further analyses. The updated data set comprises 4829 genes and 4218 isoforms;
3. Each time series was normalized by the FPKM of the GAPDH gene (dynamics almost constant, illustrated below). The unrealistically high GAPDH measurement in LH at the last time‐point suggests avoiding considering for further analysis;
4. Finally, each time‐series (genes and isoforms evolution in the four different cultures) was normalized by the FPKM value at the first time‐point (normalized FPKM), to consider only relative variations (each gene starts at normalized FPKM = 1).

Overall, the preprocessing scheme selected 3807 isoforms and 4824 genes, which will undergo further analysis. To assess the quality of the preprocessed data set, we computed the coefficient of FPKM variation (CV) for each gene and isoform.

Low‐quality bases were trimmed using erne‐filter^28^; residual adapter sequences were removed using cutadapt.^29^

Cleaned, trimmed reads were aligned against the UCSC hg19 reference human genome using tophat2.^30^ Transcript quantification based on the genome annotation was performed using cufflinks.^31^ Differential expression was performed using cuffdiff.^32^ Temporal evolution of expression was assessed by measuring the ratio of isoform expression in FPKM to the expression of GAPDH in FPKM. Expression at the first time‐point is set to 0 and the expression at following time is represented as the log2 variation of the expression relative to time 0. Genes presenting a pattern of constant up‐ or down‐regulation over time were selected as interesting candidates and plotted. Temporal evolution of ΔNp63α was also plotted.

### 2.7 Gene ontology analysis

Gene ontology (GO) enrichment analysis was performed using the Enrichment analysis tool available from the GO consortium,^33^, ^34^, ^35^ to identify biological processes significantly enriched in genes either up‐regulated or down‐regulated. To this end, the PANTHER Overrepresentation Test (Released 20,200,728) was performed using the GO Ontology database DOI: 10.5281/zenodo.4033054 Released 2020‐09‐10. Genes and isoforms either up‐regulated or down‐regulated were analysed using the Homo sapiens reference list and the GO biological process complete Annotation Data Set, using the Fisher's exact test with FDR correction.

### 2.8 Protein association analysis and druggable targets identification

Associations between up‐ and down‐regulated genes were analysed using the String suite^36^ and the following settings: network type, *full network*; meaning of network edges, *confidence*; active interaction sources, *all*; *medium confidence*. Druggable gene products were identified using the Genecards suite^37^ and by literature mining.

### 2.9 Quantitative real‐time PCR

Total RNAs from primary cell cultures were extracted and purified using the RNeasy Micro kit (Qiagen, Milan, Italy). The purity of the RNA preparation was verified by measuring its absorbance ratio at 260/280 nm. 500 ng of RNA was used to synthesize the cDNA with random hexanucleotide primers and MoMULV reverse transcriptase (Fisher Scientific, Milan,) at 42°C for 1 h. 50 ng of cDNA was amplified in an AB7900 real‐time PCR detection system (Fisher Scientific, Milan, Italy) using TaqMan™ Universal Master Mix II (Fisher Scientific, Milan, Italy), in a total volume of 20 μl. For the absolute ΔNα‐tp63 quantification (Ab‐qPCR), the level of expression of the target gene was normalized to GAPDH (Glyceraldehyde‐3‐Phosphate Dehydrogenase). For the relative gene expression analysis (Rel‐qPCR), the difference in relative target gene expression was performed using the 2 − ΔΔCt method. GAPDH was used as internal control gene. The efficiency of the target amplification (ΔNp63α) and of the reference amplification (GAPDH) were measured and found approximately equal.

### 2.10 Statistical analysis

Differences between groups were analysed by Student's *t*‐test, while multiple comparisons were performed by one‐way or two‐way anova as indicated in the text. All statistical analyses were performed using Graphpad Prism 9.0. For data shown in Figure 6, analysis of covariance was performed. The test fits independent linear models for each time series; the coefficients of the models are then compared using standard methods to identify the genes that have statistically different expression dynamics.

## 3 RESULTS

### 3.1 In vitro characterization of human primary epithelial keratinocytes reflects native and specific differences of the respective tissues of origin

We compared the proliferative potential of ESCs from different sources in vitro. To this end, human primary epithelial keratinocytes derived from skin, limbus/cornea, conjunctiva and oral mucosa were isolated (Figure 1A), cultured in vitro and serially propagated until exhaustion, with CFE being quantified at each passage. We also analysed *p63*‐defective oral mucosa ESCs obtained from three patients affected by EEC syndrome and carrying the following *p63* mutations: R279H, R304Q or R311K. Significant differences were observed between cells obtained from different tissues, and between healthy and *p63*‐defective cells. In particular, CFEs of H‐LESCs and H‐CESCs rapidly decreased after the seventh passage, and the cells could not be propagated further. Human oral mucosa stem cells (H‐OMESCs) were propagated until the 11th passage, while human skin epithelial stem cells (H‐SESCs) up to the 20th passage (Figure 1B).

**FIGURE 2:**
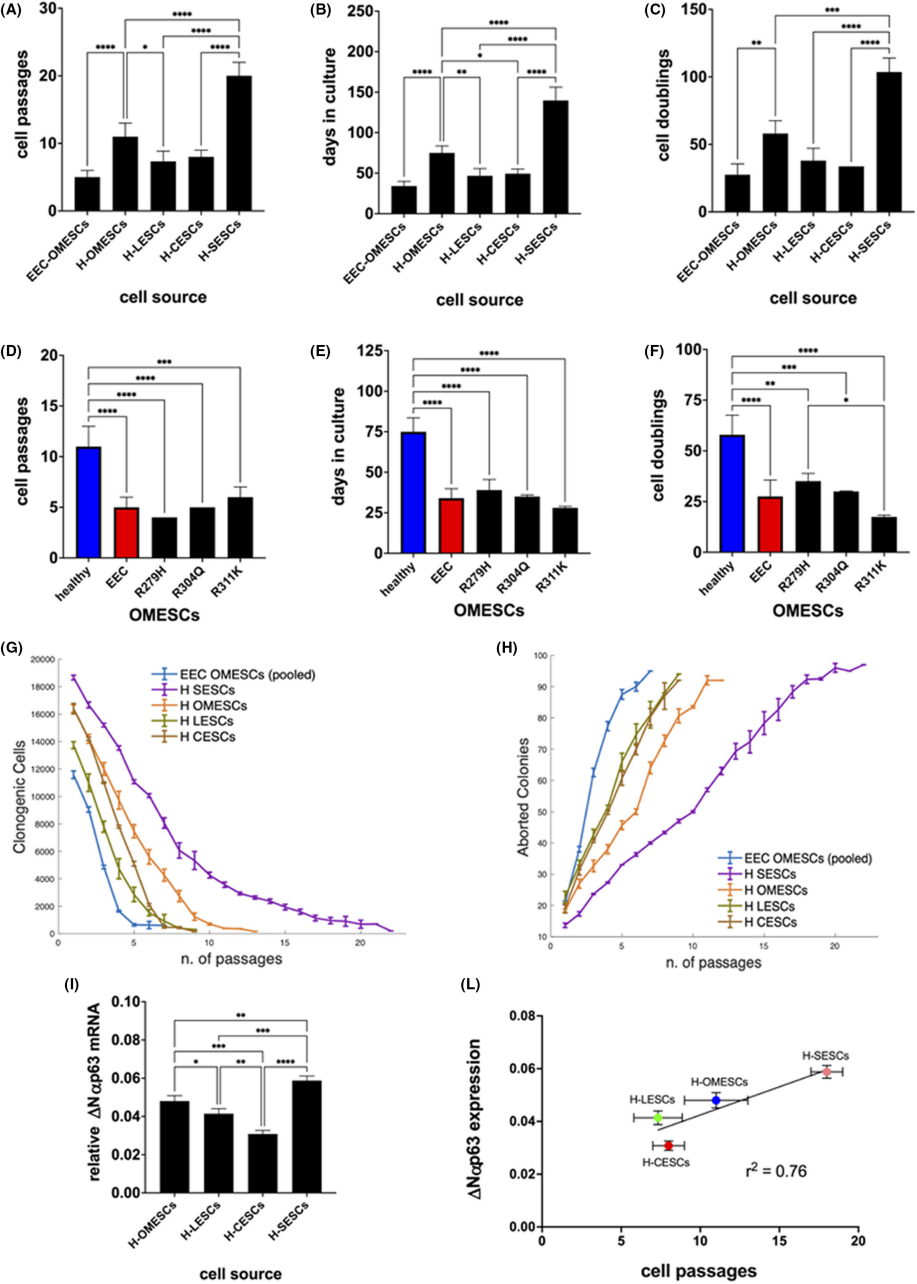
Proliferative potential of epithelial stem cells from different sources in vitro. (A) Cell passages, (B) days in culture and (C) cell doublings of EEC‐OMESCs, H‐OMESCs, H‐LESCs, H‐CESCs and H‐SESCs. (D) Cell passages, (E) days in culture and (F) cell doublings of healthy (blue) and EEC (red) OMESCs. EEC stands for the average of the values found for the three mutants R279H, R304Q and R311K OMESCs. (G) Clonogenic cell number and (H) percentage of aborted colonies relative to ESCs obtained from the indicated sources. For statistical analysis calculated by ONE way anova, please refer to Figure S1A,B. (I) Real‐time quantitative analysis of ∆Np63α expression in H‐OMESCs, H‐SESCs, H‐LESCs and H‐CESCs. Results are normalized against GAPDH. Data shown are mean + standard deviation of the mean along with the results of statistical significance as calculated by ordinary One‐Way anova with multiple comparisons (*p < .05; **p < .005; ***p < .0005; ****p < .0001). (L) The ∆Np63α expression levels of each individual ESC are plotted against the number of passages in culture. Mean values ± standard error of the means are shown, along with the chi‐squared value calculated for the linear regression. SESCs, skin epithelial stem cells; OMESCs, oral mucosa epithelial stem cells; LESCs, limbal epithelial stem cells; CESCs, conjunctival epithelial stem cells; GAPDH, glyceraldehyde 3‐phosphate dehydrogenase

In detail, H‐SESCs retained their clonogenic potential for significantly higher numbers of passages compared with ESCs from other sources, and could be maintained in culture for 20 ± 2 passages (Figure 2A). H‐OMESCs were propagated for 11 ± 2 passages, while H‐LESCs and H‐CESCs lost their clonogenic ability after 7 ± 1 and 8 ± 1 passages in culture, respectively (Figure 2A). Importantly, H‐OMESCs from EEC patients carrying the severe R311K, R304Q or R279H *p63* mutations showed a statistically significant premature decrease of their proliferative ability compared with H‐OMESCs from healthy donors (Figure 2A,D).

H‐LESCs, H‐CESCs and *p63*‐mutated OMESCs displayed a shorter lifespan and could be maintained in vitro for less than 50 days (46 days on average for H‐LESCs, 49 for H‐CESCs, 39 for R279H‐OMESCs, 35 for R304Q‐OMESCs and 28 for R311K‐OMESCs), compared with 75 days for H‐OMESCs and 139 for H‐SESCs (Figure 2B,E).

The mean of cumulative cell doublings confirms the results described above. In fact, H‐SESCs can exceed 100 cell doublings and H‐OMESCs can easily reach 60 cell doublings. On the contrary, H‐LESCs and H‐CESCs reached the end of their lifespan with a mean of 37 and 39 cumulative cell doublings, respectively, while R279H‐, R304Q‐ and R311K‐OMESCs after only 34, 29 and 17 doublings, respectively (Figure 2C,F).

The reductions in CFE of H‐LESC, H‐CESC and *p63*‐defective H‐OMESC cultures were faster compared with what observed with H‐SESCs and H‐OMESCs at the same cell passages, with the latter (H‐SESCs and H‐OMESCs) maintaining higher numbers of clonogenic cells also at later cell passages (Figures 2G and S1A for statistical analysis). At passage 6, when H‐LESCs, H‐CESCs and *p63*‐defective OMESCs were almost exhausted, H‐OMESCs and H‐SESCs retained on average 5.600 and 10.000 clonogenic cells, respectively. In particular, H‐SESCs and H‐OMESCs had 50% of aborted colonies after 10 and 6 passages, respectively, while H‐LESCs and H‐CESCs reached this percentage already at the fourth passage in culture (Figures 2H and S1B for statistical analysis).

The expression of the ΔNp63α marker followed the same trend and showed a significantly higher expression in H‐SESCs compared with the other cultures, thus highlighting the different regenerative properties of ESCs obtained from different sources (Figure 2I). Interestingly, the expression levels of ΔNp63α at the first passage positively correlated with the number of passages that cells are able to perform during serial passages (Figure 2L), thus predicting the lifespan length of SCs.

### 3.2 DAPT administration extends the lifespan of stem cells

**FIGURE 3:**
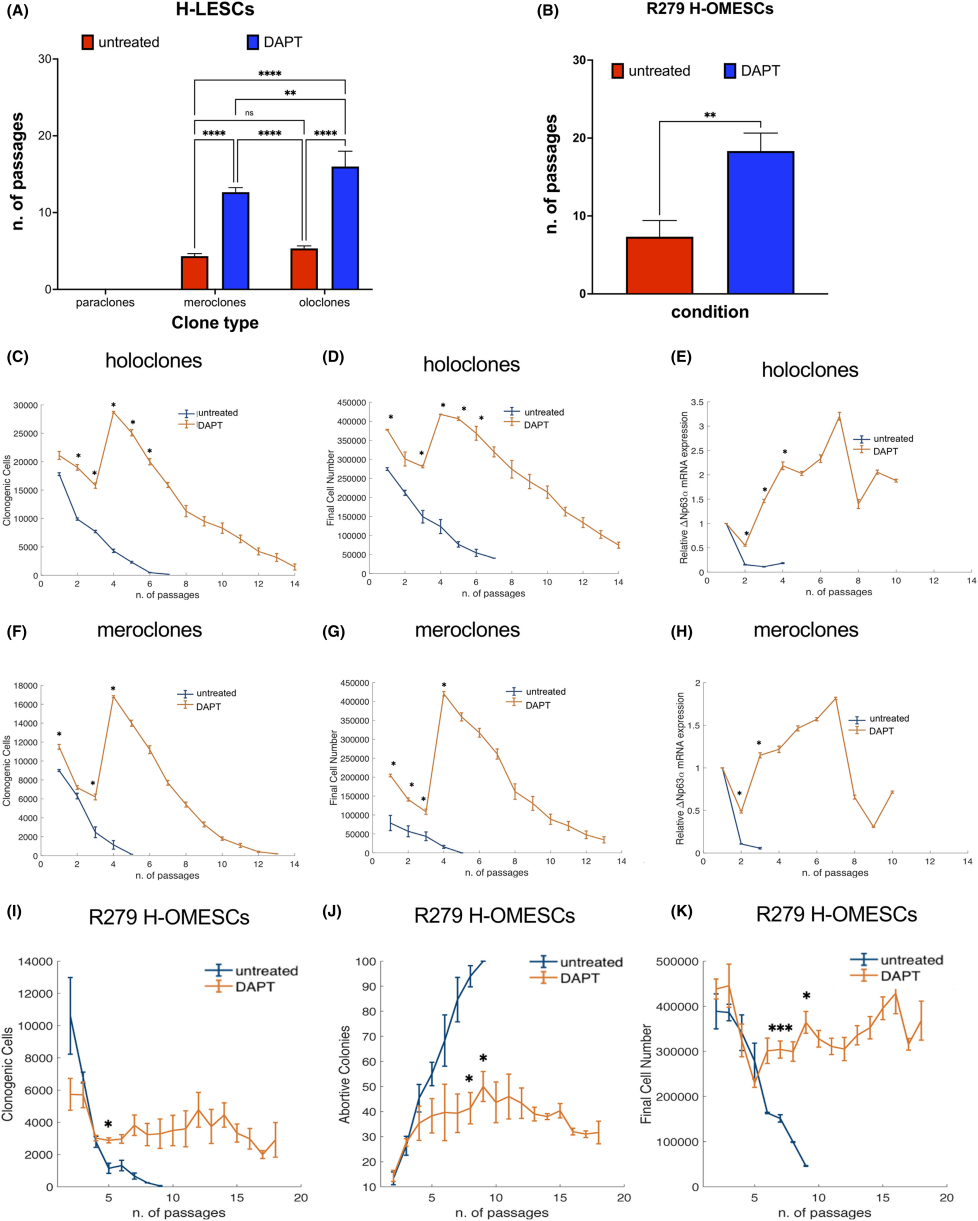
DAPT administration extends the lifespan of stem cells. Cell cultures derived from holoclones and meroclones obtained from donor limbus and EEC‐OMESCs were treated with DAPT. The number of passages in the absence (red columns) or presence (blue columns) of DAPT (A). Significant increase in the number of passages after DAPT treatment of EEC‐OMESCs (B). Clonogenic cells, final cell number and ∆Np63α expression quantified by qPCR from holoclones (C–E), meroclones (F–H) and R279 H‐OMESCs (I–K) either untreated or treated with DAPT. Data are shown as mean ± standard deviation along with the results of statistical significance as calculated by ordinary one‐way anova with multiple comparisons (*p < .05; **p < .005; ***p < .0005; ****p < .0001). EEC‐OMESCs: ectrodactyly‐ectodermal dysplasia‐clefting oral mucosa epithelial stem cells; DAPT: N‐[N‐(3, 5‐difluorophenacetyl)‐l‐alanyl]‐S‐phenylglycine t‐butyl ester

To delay cellular senescence, we treated cell cultures derived from holoclones, meroclones and paraclones obtained from donor limbus with N‐[N‐(3,5‐difluorophenacetyl)‐l‐alanyl]‐S‐phenylglycine t‐butyl ester (DAPT, γ‐secretase inhibitor and indirect Notch signalling inhibitor). Consistent with the key role of Notch in mediating the differentiation of adult ESCs, the continuous administration of DAPT led to an enrichment of ESCs as well as an extension of their lifespan. As expected, the effect was proportional to the initial ESC content. DAPT treatment significantly increased the lifespan of both holoclones (from 5.3 to 16 passages; *p* < .0001) and meroclones (from 4.3 passages to 12.7 passages; *p* < .0001), compared with untreated controls (Figure 3A). However, DAPT had no effects on cells derived from paraclones, which are comprised exclusively of terminally differentiated cells, and could not be propagated in culture (Figure 3A).

Importantly, DAPT administration also significantly increased the proliferative potential of H‐OMECS derived from three EEC patients carrying the mutation R279H. Untreated cells could be maintained in culture for 7 ± 2 passages, whereas DAPT‐treated cells for 18 ± 2 passages (Figure 3B; *p* = .0036).

DAPT‐treatment increased the number of clonogenic cells from 4.300 to 28.700 for holoclones and from 1.140 to 16.800 for meroclones (Figure 3C,F) at passage 4. Similarly, the number of final cells followed the same trend, rising from 123.500 to 418.000 for holoclones and from 32.000 to 420.000 for the cultures derived from meroclones (Figure 3D,G) at passage 4. Such results were confirmed by higher and more sustained expression of the ESC marker ΔNp63α, for both cultures derived from holoclones and meroclones upon DAPT treatment, as assessed by q‐PCR (Figure 3E,H).

**FIGURE 4:**
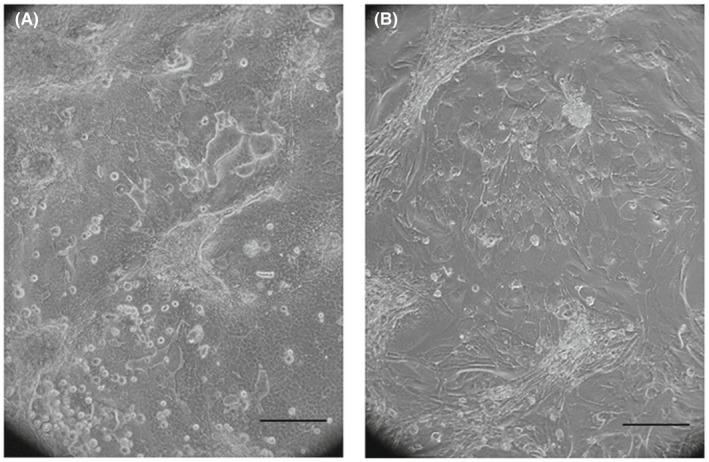
Changes in cell morphology following DAPT discontinuation. Cells cultured in medium containing DAPT show a stem‐like morphology (A). DAPT removal from culturing medium leads to morphological changes compatible with senescence (B). Scale bar: 100 μm. Abbreviations: DAPT: N‐[N‐(3, 5‐difluorophenacetyl)‐l‐alanyl]‐S‐phenylglycine t‐butyl ester

In addition, DAPT treatment significantly affected the number of clonogenic cells (from 1.315 to 2965 at passage 5), percentage of abortive colonies (68% and 40% at passage 5, respectively) and final cell number (from 163.000 to 300.000 at passage 5) of R279 H‐OMESCs (Figure 3I–K). Overall, these data strongly support the ability of DAPT to extend the lifespan of ESCs. Removal of DAPT at passage XV from cell cultures (defined as *switch* throughout the text) resulted in a rapid change in morphology, with cells appearing as usually observed in end‐stage cultures: in fact, they could only be cultured for two more passages, thus excluding any immortalization process being induced following DAPT administration (Figure 4A,B).

### 3.3 Karyotype analysis, telomere length and mitochondrial activity throughout DAPT induced proliferation

**FIGURE 5:**
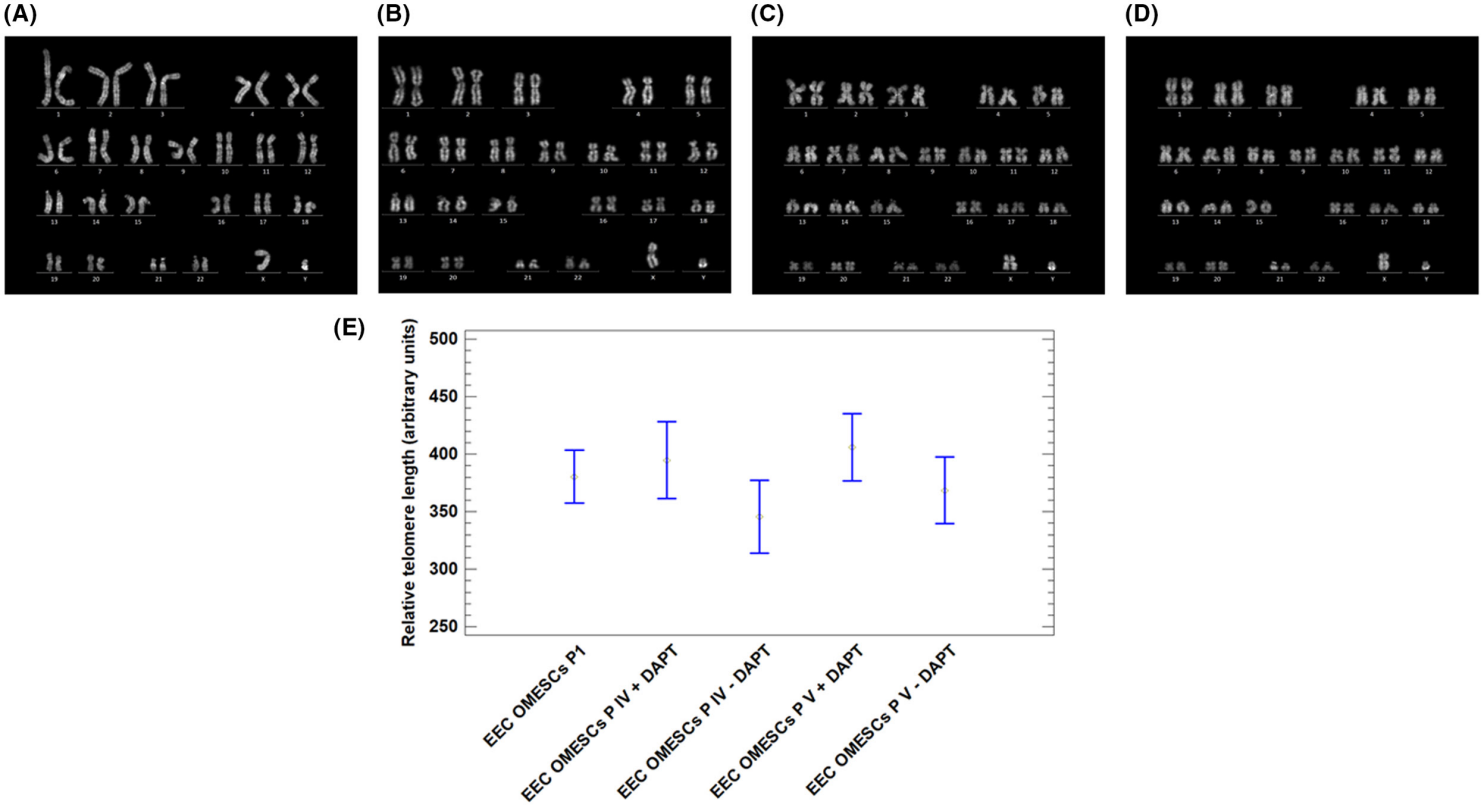
DAPT induces telomere elongation without altering the karyotype. Representative pictures displaying EEC OMESCs karyotype. (A) EEC‐OMESCs karyotype at passage I, (B) EEC‐OMESCs + DAPT karyotype at passage IV, (C) EEC‐OMESCs + DAPT karyotype at passage VII, (D) EEC‐OMESCs + DAPT at passage XVII. In (E), bar plot comparing the relative telomere length in EEC‐OMESCs pI, EEC‐OMESCs + DAPT pIV, EEC‐OMESCs + DAPT pVII, EEC‐OMESCs + DAPT pXVII. EEC‐OMESCs: ectrodactyly‐ectodermal dysplasia‐clefting oral mucosa epithelial stem cells; DAPT: N‐[N‐(3, 5‐difluorophenacetyl)‐l‐alanyl]‐S‐phenylglycine t‐butyl ester

To assess any possible transformation of cells following DAPT administration, we performed a karyotype analysis on EEC‐OMESCs at different passages in culture (pIV, pVII, pXVII). The cytogenetic assay confirmed that no numerical or structural chromosomal abnormalities were observed compared with cells cultivated without DAPT (i.e., cells at passage pI, the initial and, therefore, standard of reference for lifespan analysis) (Figure 5A–D).

Q‐FISH analysis of the telomeres of EEC‐OMESCs showed increased telomere length in cells grown with DAPT (p IV, pV), compared with the same cells cultivated without DAPT (Figure 5E). These results correlate well with the extended lifespan observed in SCs treated with continuous administration of DAPT, thus confirming that elongation of telomeres is linked to increased cellular half‐life.^38^

Since the senescence process in vitro is likely to involve both telomeres and mitochondria,^39^ we evaluated the mitochondrial activity in EEC‐OMESCS after DAPT administration by means of a MitoTracker assay, and analysed the signal of probes, which passively diffuse across the plasma membrane and accumulate in active mitochondria. As shown in Figure S2, the mitochondrial staining (in red) is not detectable in EEC‐OMESCs at passage I (A), passage VI with DAPT (B) and passage XVII with DAPT (D), while it is detectable in cells at passage VI not treated with DAPT (C) and at passage XVII after removal of DAPT at passage XV (*switch*) (E). Quantification of mitochondrial activity is shown in Figure S2F.

Because a low mitochondrial activity has been previously reported to be linked to a higher SC potential in ex vivo experiments,^40^ our results seem to confirm that the administration of DAPT enriches the SC population, as shown by the low mitochondrial activity of treated cell, and delayed premature senescence.

### 3.4 Comparative epithelial stem cell transcriptomics analysis

To investigate the transcriptional changes occurring in ESCs during their life time, we performed RNAseq analysis in four cell culture samples [(i) Limbal Holoclone (LH) cultures, (ii) LH cultures after DAPT treatment (LH‐DAPT), (iii) H‐OMESCs from healthy donors and (iv) EEC‐OMESCs] at three stages of the lifespan, selected on the basis of the clonogenic ability and proliferative potential data, as shown in Figure 2. The initial stages of the lifespan were passage 2 for H‐OMESCs and EEC‐OMESCs, and passage 3 for LH and LH‐DAPT cultures. Intermediate stages were passage 4 for H‐OMESCs, passage 3 for EEC‐OMESCs, passage 4 for LH and passage 6 for LH‐DAPT cultures. The terminal stages were passage 7 for H‐OMESCs, passage 5 for EEC‐OMESCs, passage 5 for LH and passage 12 for LH‐DAPT cultures. An average of 32.7 million of quality‐filtered reads for samples were generated. A total of 49.772 isoforms and 27.028 genes were investigated to highlight a common age‐dependent regulation across samples. The identification of significant genes/isoforms was based on the q‐value (adjusted p‐value for false discovery rate) criterion.

**FIGURE 6:**
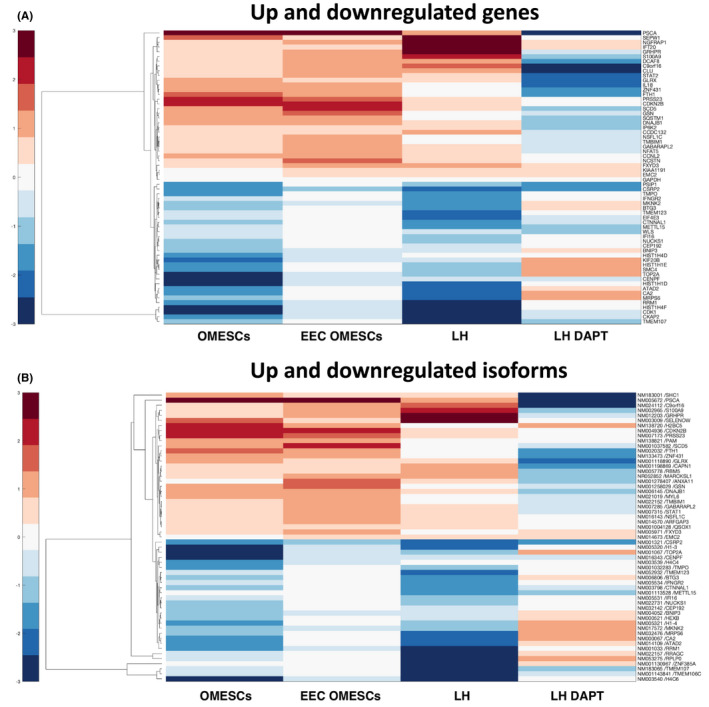
Comparative epithelial stem cells transcriptomics analysis. Heatmaps depicting mean of ex vivo gene expression data sets, representing (A) genes and (B) isoforms negatively correlated with lifespan and up‐regulated during senescence and vice versa (red: high values, blue: low values)

The RNAseq analysis revealed that the expression of 61 genes and 20 isoforms was found deregulated during cell senescence, as shown in the heatmap (Figure 6A,B). The differential expression analysis of ΔNp63α isoform reveals a typical trend of decline during the differentiation process (Figure S3). This isoform showed a strong decrease in LH samples during cell culture passages that appeared less evident in OMESCs and EEC‐OMESCs samples. Differently, an initial overexpression was observed in LH/LH‐DAPT samples, followed by a progressive and constant long‐term maintenance with a less steep drop in the latest stages. Interestingly, a comparable profile was observed by analysing the expression of *WLS* (WNT Ligand Secretion Mediator), essential for WNT (Wingless/Integrated) protein secretion, and SC population maintenance, The graph‐based gene expression profiles of all genes and isoforms are shown in Figures [Link], [Link]. The differential expression analysis reveals two strong trends, a first one represented by genes negatively correlated with lifespan and up‐regulated during senescence and a second one by genes positively correlated with lifespan and down‐regulated during senescence.

**FIGURE 7:**
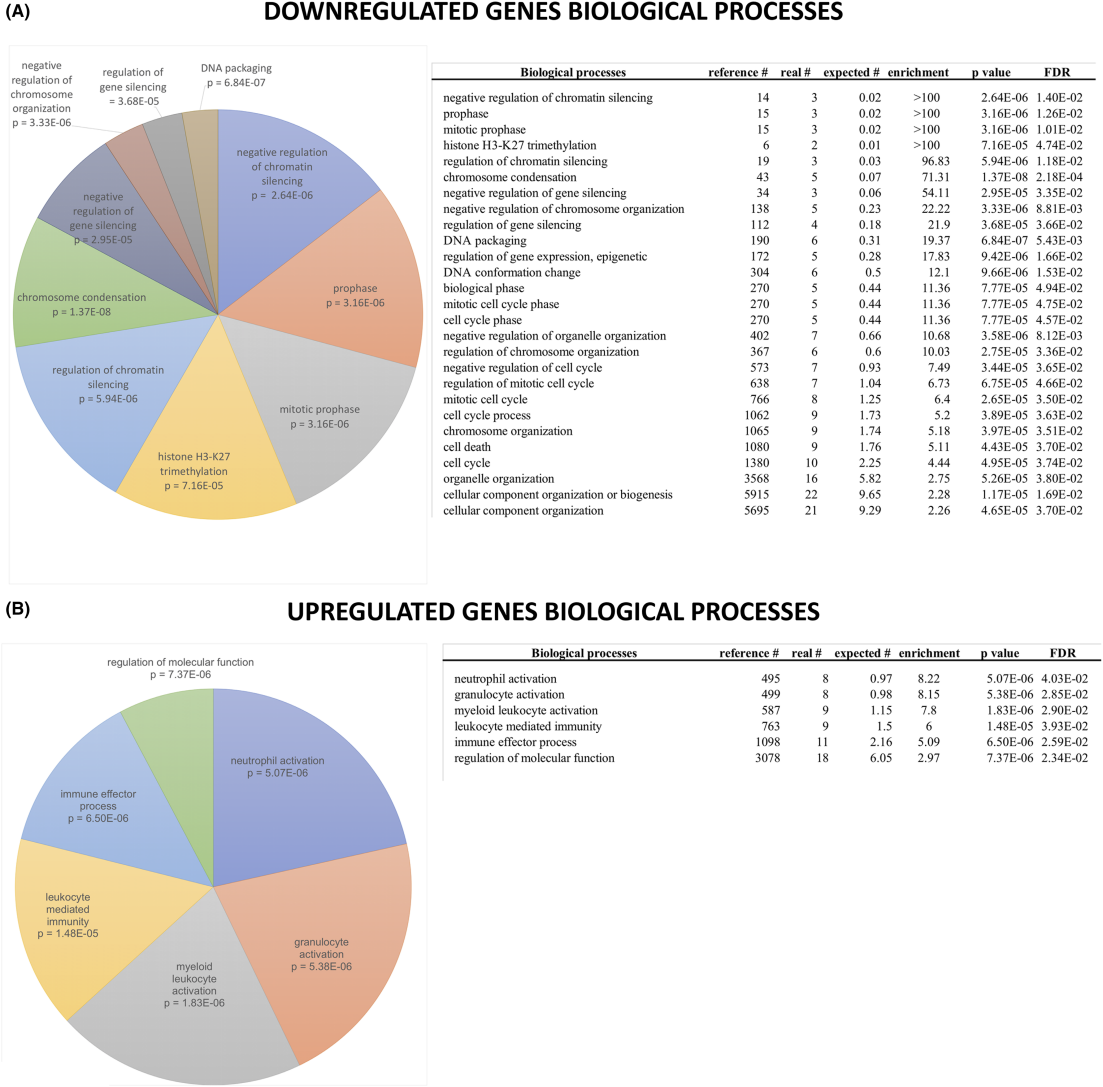
Gene ontology (GO) enrichment analysis of deregulated genes in epithelial SCs. Genes either down‐regulated (A) or up‐regulated (B) during SC lifespans were used to perform GO enrichment analysis as described in the materials and methods section. All significantly enriched biological processes are shown on the left: the size of each slice is proportional to the enrichment of each biological process with respect to the reference list, and the *p* value is indicated. All other relevant information is reported in the right panels

Comparative RNAseq analysis revealed a significant differential gene expression during the lifespan of cells. In total, 31 genes and 11 isoforms were up‐regulated in the last passages of the lifespan (Table S1), whereas 30 genes and 8 isoforms were consistently expressed at higher levels in the first passages, thus suggesting a potential down‐regulation with senescence (Table S2). We further performed a GO enrichment analysis on the up‐regulated and down‐regulated gene lists reported in Tables S1 and S2, to identify biological processes involved in ESC senescence. We could identify 27 biological processes associated with down‐regulated genes (Figure 7A) and 6 associated with up‐regulated genes (Figure 7B). Our results indicate that SC senescence is associated to a down‐regulation of cellular products mainly associated with “negative regulation of gene silencing”, “prophase”, “mitotic prophase”, “histone H3‐K27 trimethylation” and “regulation of chromatin condensation” (all with an enrichment >95x). On the other hand, up‐regulated genes were mainly linked to inflammation and immune response.

### 3.5 Analysis of marker expression during lifespan

**FIGURE 8:**
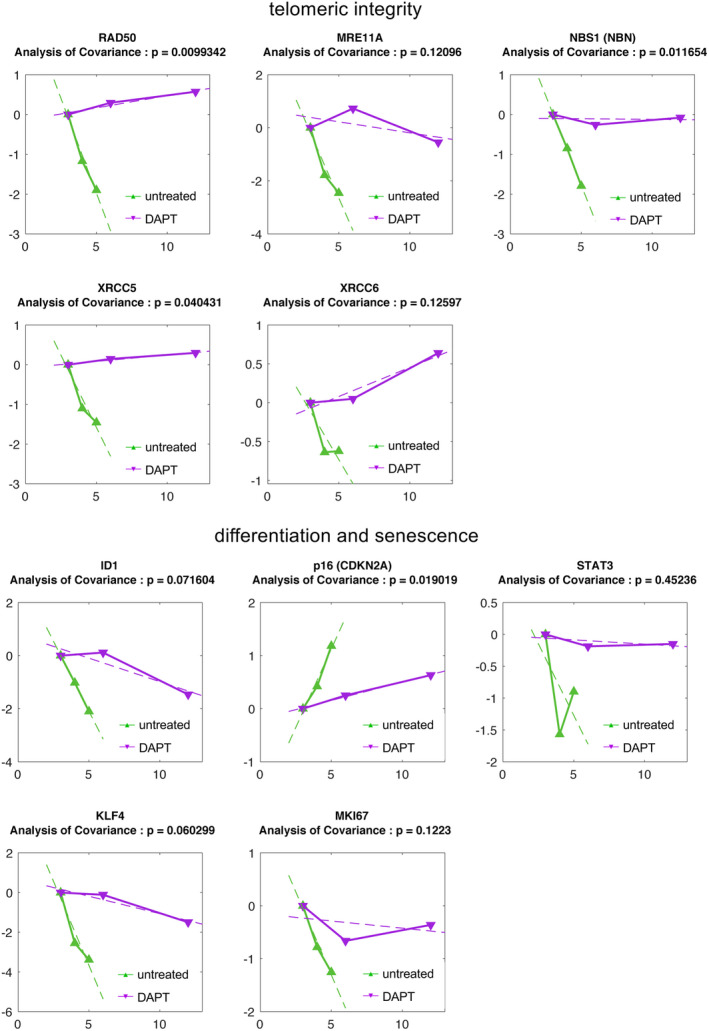
Expression profiles of senescence markers. The expression profiles of a panel of genes involved in the maintenance of telomeric integrity and/or associated with differentiation and senescence in keratinocyte stem cells were obtained from Limbal Holoclone cultures either untreated (LH) or treated with DAPT (LH‐DAPT) by means of RNAseq analysis. Normalized expression of the indicated markers is plotted against the culture passage. The time series relative to LH and LH‐DAPT is compared using an analysis of covariance test (*p* < .05). LH, limbal holoclones

We extrapolated the expression profiles of a panel of genes involved in the maintenance of telomeric integrity (*RAD50*, *MIRE11A*, *NBS1*, *XRCC5* and *XRCC6*), and/or associated with differentiation and senescence in keratinocyte SCs (*ID1*, *p16*, *STAT3*, *KLF4* and *MKI67*). RNAseq data obtained for LH and LH‐DAPT cultures are reported in Figure 8. In particular, we observed that *RAD50*, *MRE11* and *NBS1* (associated to the MRN complex involved in the detection and the repair of DNA double strand breaks), and *XRCC5* and *XRCC6* (forming the Ku heterodimer, involved in the repair of double strand breaks) showed a more stable expression over time, following treatment with DAPT. Such genes are vital for the maintenance of telomere length and have been linked to cellular senescence. The expression levels of both *Ku70* and *MRE11* were negatively correlated with cell senescence.^41^, ^42^, ^43^ Moreover, DAPT‐treated cells showed a slower decrease in the expression levels of Id‐1 of helix–loop–helix protein, linked to the immortalization of human primary keratinocytes. This is possibly due to a partial inhibition of *p16* (*CDKN2A*) expression,^44^ since keratinocytes undergoing senescence strongly induce *CDKN2A* expression.^45^ Accordingly, *CDKN2A* increase was slower in DAPT‐treated cells compared with untreated ones. On the other hand, STAT‐3 transcripts dropped rapidly in LH samples, while remained constantly elevated in LH‐DAPT. STAT‐3 is a transcription factor activated by a variety of growth factors and cytokines and plays important roles in cell growth and survival. STAT3 activation, triggered by continued exposure to growth factors stimulation, has been demonstrated to promote escape from senescence checkpoints, thus contributing to protection from cellular senescence.^46^ Krüppel‐like factor 4 (KLF‐4) has been recently recognized as a regulator of keratinocyte senescence: this transcription factor is important in sustaining keratinocyte proliferation and its silencing seems to be sufficient to trigger cellular senescence.^47^ According to our RNA‐seq data set, *KLF‐4* levels rapidly drop during lifespan of untreated cells, while DAPT exposure slows down its decrease. Finally, *MKI67* is an established genetic marker for cellular proliferation in keratinocyte. It seems to be strongly down‐regulated in LH cultures, as expected, while the initial expression levels are maintained during the different passages in cells treated with DAPT, thus confirming that the senescence process is delayed in this culture.^48^ Overall, these data confirm that following treatment with DAPT, ageing process of LH cultures seems to slow down.

### 3.6 Identification of druggable protein networks involved in epithelial stem cell senescence

**FIGURE 9:**
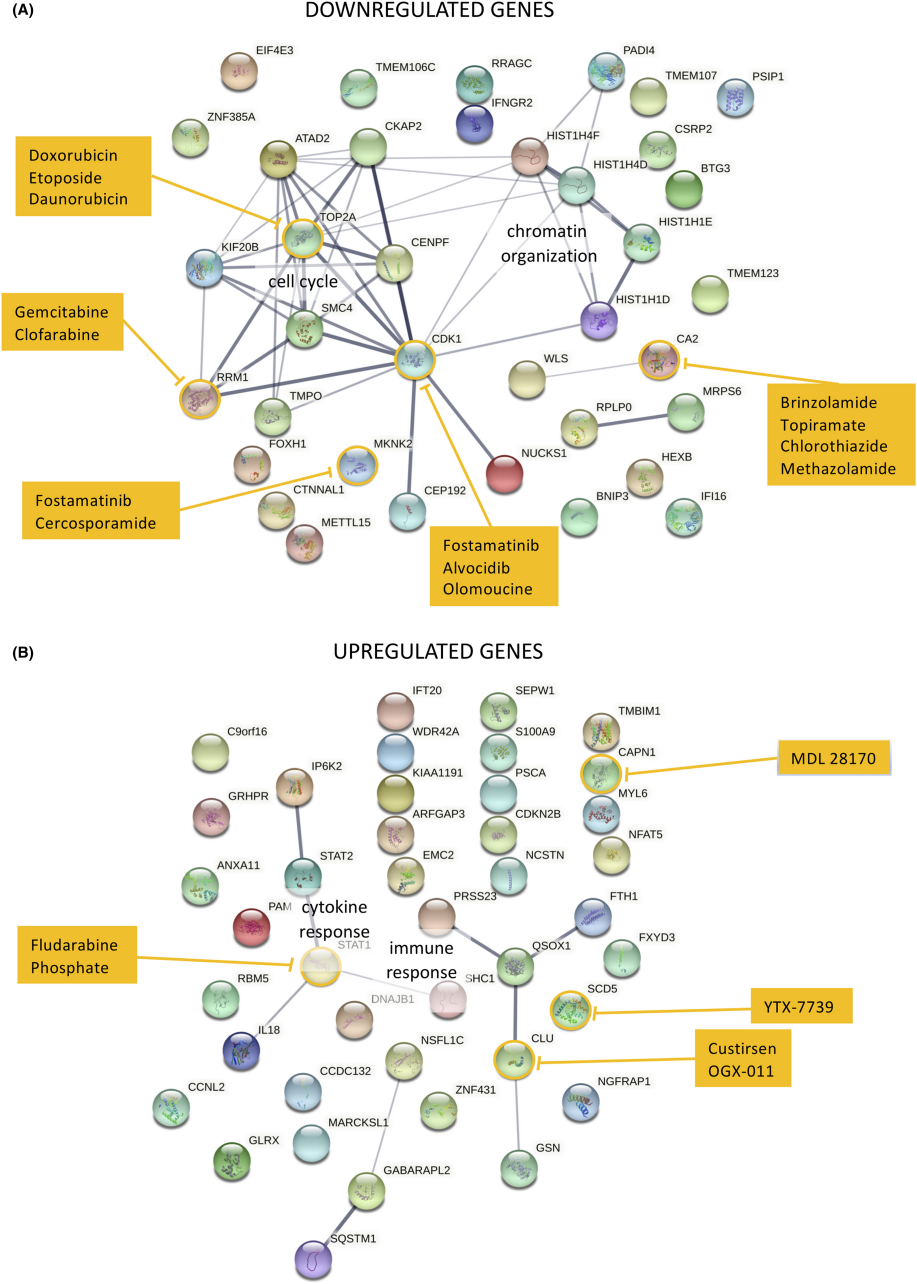
Identification of druggable targets involved in SCs ageing and their connections. Genes that are either down‐regulated (A) or up‐regulated (B) were used to reconstruct their potential connections using String software and drugs targeting them were retrieved from the Genecards database. Line thickness is proportional to the levels of confidence, druggable gene products are circled in yellow and potential drugs targeting such genes are listed in the yellow boxes

Because our data showed that it is possible to modulate the senescence of ESCs pharmacologically by continuous administration of DAPT, we sought to identify additional drugs capable of further modulating such process. To this end, we reconstructed a protein networks based on the RNAseq data and identified potential drugs able to modulate the senescence process based on the information available on the Genecard suite (Figure 9). Our results suggest that five down‐regulated and four up‐regulated gene products are targetable by 19 different approved or experimental drugs. Among down‐regulated gene products, CDK1 and TOP2A, associated with 10 and 7 other factors, respectively, appeared the most central druggable factor (Figure 9A). Among druggable up‐regulated factors, STAT1 was associated with three additional gene products (Figure 9B).

## 4 DISCUSSION

The functional decline of SCs has been associated with many age‐related conditions and diseases. Previous reports have shown that attenuation/reversal of the age‐induced functional decline of SCs can be achieved through modulation of the environment, thus leading to the hypothesis that ageing of SCs occurs largely at the epigenetic level.^49^

In the first part of this study, we have focused on the comparative features of SCs obtained from four different epithelial tissues. Our results suggest that the in vitro behaviour of ESCs reflects their in vivo physiology. We show that keratinocyte SCs have an intrinsically limited and lineage‐specific lifespan. Thus, as the human epidermidis is renewed monthly,^50^ in vitro proliferation of H‐SESCs proceeds for 20 days. The proliferation ability of SCs potential could notably increase thanks to administration of the Notch signalling inhibitor DAPT, which could enrich the SCs population obtained from small biopsies and delay their premature senescence.

The ability of DAPT to delay cell senescence, and therefore extend the replicative properties, was also tested on oral mucosa ESCs derived from three EEC patients (R279H‐OMESCs). It is known that cells from these patients are affected by premature senescence. Interestingly, the addition of DAPT increased the number of cell passages in vitro from 7 ± 2 to 18 ± 2 i.e., about three times more than the normal length of the lifespan of these cell cultures. The analysis of the karyotypes of EEC‐OMESCs treated with DAPT did not reveal any numerical or structural chromosomal abnormalities. However, both the telomere length and the number of passages in culture were increased, thus confirming that the elongation of telomeres is associated with an increased half‐life of the cells (27). Our preliminary findings would therefore indicate that the use of DAPT could slow down the senescence of epithelial SCs from patients with EEC syndrome, thus suggesting interesting pharmacological opportunities for the treatment of these patients.

Our data show a differential longevity of the analysed epithelia in vitro, greater for the skin and lower for the cornea (Figure 2), which is consistent with the extension and the physiological function of the tissue.^51^ Accordingly, the limbus has a smaller number of SCs than its neighbouring conjunctiva, which requires more SCs for normal renewal as having a larger extension. While the niches of the cornea are located in a specific and delimited area (the limbus) in which the palisades of Vogt are organized to protect the SCs, the distribution of the niches in the skin encompass the entire basal layer. This is likely due to the epithelium of the skin being constantly subjected to stress and continuously renewed.^52^

In our previous studies,^19^, ^20^, ^21^ we provided evidence that a major factor in the pathophysiology of EEC syndrome is the premature degeneration of the limbal epithelial SCs and the palisade structures. Indeed, the inability of p63 defective‐limbal SCs to generate a fully stratified corneal epithelium leads to a “*continuous activation status*”, causing their rapid exhaustion and thus premature senescence in vivo. All EEC mutants showed defects in stratification and differentiation and shorter lifespans in vitro.^20^, ^21^ Nevertheless, in this study we demonstrate that H‐LESCs potency and length of lifespan are at least three times lower than H‐SESCs (Figure 2), a likely explanation of the early signs of degeneration seen in the ocular surface of patients with EEC syndrome.

In the second part of the work, the transcriptional changes occurring in keratinocyte SCs during senescence were investigated by means of RNAseq in four distinct sample collections (LH, LH after DAPT, H‐OMESCs, EEC‐OMESCs). We identified several genes strongly related to senescence. In our model, the starting point of cellular differentiation and senescence involves the alterations in gene expression due to chromatin changes. A tightly packaged chromatin structure reduces the access to DNA, thus limiting inappropriate gene expression and genomic instability. Instead, open chromatin structures lead to unregulated gene expression and genomic instability.^53^ Consistent with this, many chromatin‐remodelling enzymes have been identified with key roles in differentiation and development. The diminished expression of *ATAD2, CENPF, CEP192, HIST1H1D, HIST1H1E, HIST1H4D, HIST1H4F, NUCKS1, SMC4, TMPO, TOP2A* correlates with age and a general loss of histones coupled with local and global chromatin remodelling, an imbalance of activating and repressive histone modifications.^54^

The diminished expression of *BNIP3, BTG3, TMPO, CDK1, CKAP2, CSRP2, EIF4E3, IFI16, IFNGR2, KIF20B, METTL15* and *RRM1* correlates extensively with their role in cell proliferation and survival, as well as the increased expression of *CCNL2, CDKN2B, CLU, IP6K2, PSCA, S100A9*, all involved in cell cycle and proliferation.^49^, ^55^

Some of the up‐regulated genes show a strong correlation with senescence and include:

- *NCSTN*, a critical component of the γ‐secretase complex involved in the Notch and Wnt signalling cascades, fundamental to induce the cellular differentiation or cell apoptosis and up‐regulated with ageing^56^;
- *CAPN1*, implicated in various ageing phenomena and diseases of late life, including cataract formation, erythrocyte senescence, diabetes mellitus type 2, hypertension, arthritis, and neurodegenerative disorders^57^;
- *CLU*, a molecular chaperone that increases stress resistance and significantly extends lifespan, when overexpressed^58^;
- *SEPW*, a selenoprotein involved in redox‐related processes, muscle growth and differentiation, and in the protection of neurons from oxidative stress during neuronal development^59^;
- *STAT1*, a signal transducer and transcription factor playing an essential role in response to interferon (IFN) signalling and regulating many cellular processes, such as proliferation, differentiation and cell death.^60^, ^61^

Among the down‐regulated genes, we found the following ones to be interesting:

- *TOP2A*, a topoisomerase involved in regulating the topological states of DNA, has been indirectly linked to Werner syndrome ATP‐dependent helicase (WRN), a gene responsible for Werner's syndrome, a human disorder causing symptoms of accelerated ageing in early adulthood^62^;
- *CDK1*, an important regulator of the cell cycle, also appears to be involved in apoptosis. Age‐related alterations to CDK1 have been reported in rats.^63^

Dysfunctional regulation of mitochondrial processes contributes to oxidative stress and cell death during ageing. With advancing age, all the mitochondria of a cell may be damaged, thus causing significant harm. The decline in mitochondrial turnover caused by reduced biogenesis and inefficient degradation seems to be a particularly crucial factor in ageing.^64^ At least four genes involved in mitochondrial metabolism were found to be down‐ (*BNIP3*, *METTL15*, *MRPS6*) or up‐regulated (*GABARAPL2*), respectively. The *MRPS6* gene encoding a mitochondrial ribosomal protein, previously linked to ageing and longevity in model organisms (i.e., mice, *Caenorhabditis elegans* ^65^), results to be down‐regulated. The low mitochondrial metabolism detected in cells treated with DAPT confirm that the administration of DAPT enrich the SCs population, and delay the premature senescence.

Eight differentially expressed genes are directly related to autophagy and cell death processes: *GABARAPL2, SQSTM, NGFRAP1, TMBIM1, RBM5* and *SHC1* (up‐regulated), *TMEM123* and *TMEM106C* (down‐regulated).

Finally, numerous gene involved in regulatory processes important for development, survival, proliferation and differentiation were found up‐ (*KIAA1191*, *S100A9*, *SEPW1* and *TMBIM1*) or down‐regulated (*CSRP2*, *IFI16* and *PSIP1*).

## 5 CONCLUSIONS

In the current study, we provided some insights into the self‐renewal and senescence features of epithelial tissues. We show that the in vitro proliferative capacities of epithelial SCs from skin, cornea, conjunctiva and oral mucosa tissues, are different and lineage‐specific and reflect the different physiological needs and characteristics of each tissue. We provide evidence that lifespan may represent a valid model for studying ageing and senescence features in vitro and that it can be extended with the help of suitable pharmacological inhibitors. Moreover, we report a list of genes, the expression of which strongly changes during SC lifespan. Additionally, we show that DAPT treatment can slow the senescence process by extending the replicative capacity of SCs, further confirming the importance of Notch signalling in ageing.^66^ Finally, we identified a number of approved or experimental drugs targeting several factors either up or down‐regulated during SC lifespans even in the presence of DAPT, implying the possibility of further manipulate ESCs proliferative potential downstream of Notch signalling (Figure 9). These data will be useful to expand our knowledge on the genetic basis of senescence, and, possibly, pave the way to the pharmacological modulation of the ageing process of ESCs.

## AUTHOR CONTRIBUTIONS

**Vanessa Barbaro:** Conceptualization (equal); data curation (equal); supervision (equal); validation (equal); writing – original draft (equal); writing – review and editing (equal). **Antonio Orvieto:** Methodology (equal); software (lead); validation (supporting). **Gualtiero Alvisi:** Conceptualization (equal); data curation (equal); formal analysis (equal); writing – review and editing (equal). **Marina Bertolin:** Data curation (equal); methodology (equal). **Filippo Bonelli:** Data curation (equal). **Thomas Liehr:** Data curation (equal); formal analysis (equal). **Tigran Harutyunyan:** Formal analysis (equal); funding acquisition (equal). **Stefanie Kankel:** Data curation (equal). **Gordana Joksic:** Data curation (equal); funding acquisition (equal). **Stefano Ferrari:** Validation (equal); writing – review and editing (equal). **Elena Daniele:** Methodology (equal). **Diego Ponzin:** Validation (equal); writing – review and editing (equal). **Daniela Bettio:** Formal analysis (equal); funding acquisition (equal). **Leonardo Salviati:** Validation (equal); writing – review and editing (equal). **Enzo Di Iorio:** Conceptualization (equal); formal analysis (equal); funding acquisition (lead); project administration (lead); supervision (lead); validation (equal); writing – original draft (equal); writing – review and editing (lead).

## CONFLICT OF INTEREST

The authors confirm that there are no conflicts of interest.

## Supporting information


Figure S1

Figure S2

Figure S3

Figure S4

Figure S5

Figure S6

Figure S7

Table S1

Table S2

## Data Availability

The data that support the findings of this study are available from the corresponding author upon reasonable request.
