# Supplementary figures and images for: Analysis and pharmacological modulation of senescence in human epithelial stem cells
Source: J Cell Mol Med. 2022 Jun 15;26(14):3977–94. doi: 10.1111/jcmm.17434 (PMC9279594; doi:10.1111/jcmm.17434)

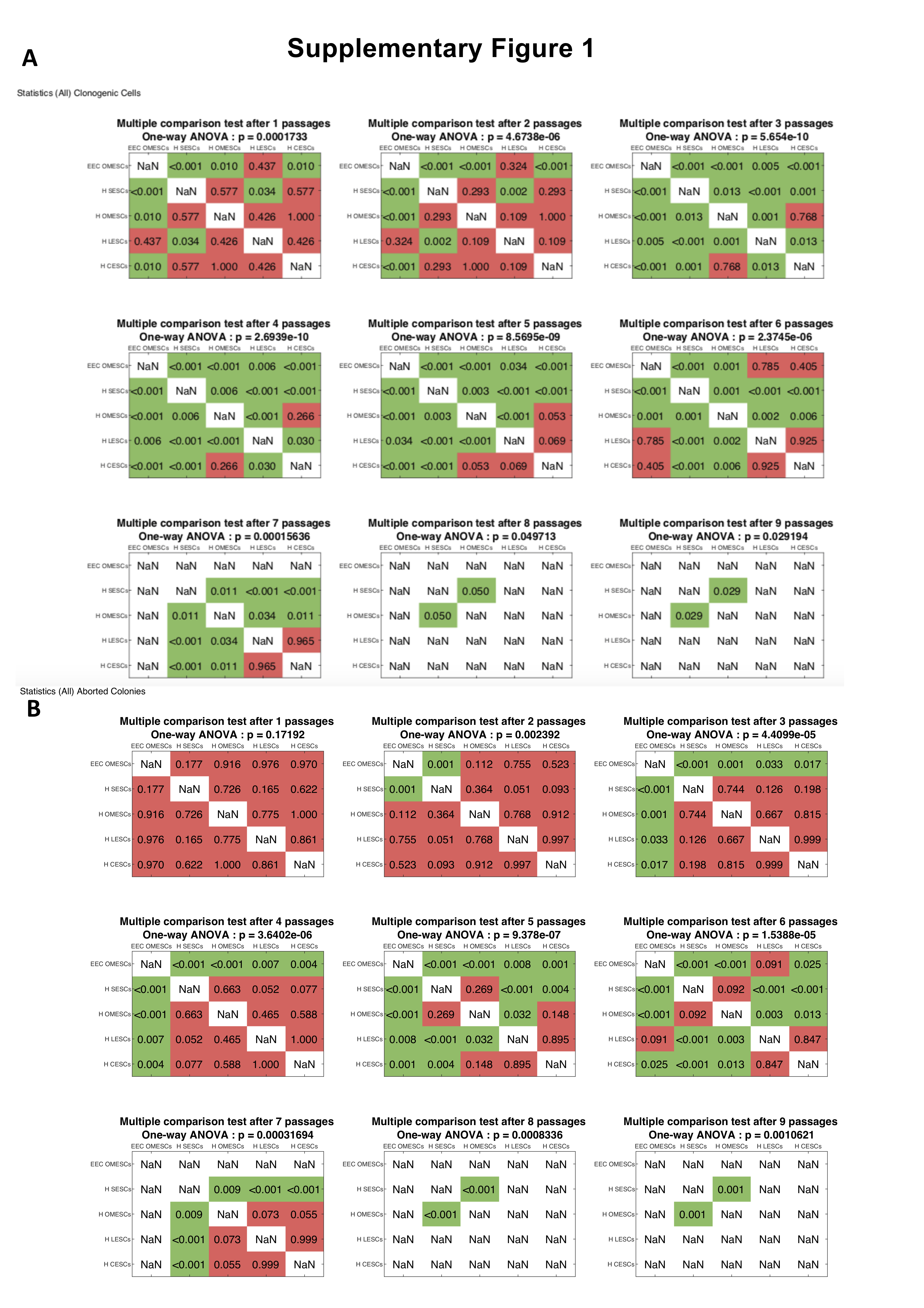

Supplement: Supplementary file 1 — Figure S1 [file JCMM-26-3977-s006.tiff]

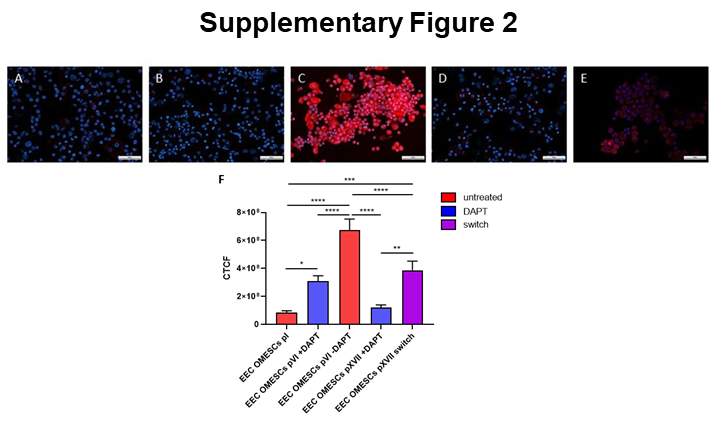

Supplement: Supplementary file 2 — Figure S2 [file JCMM-26-3977-s004.tif]

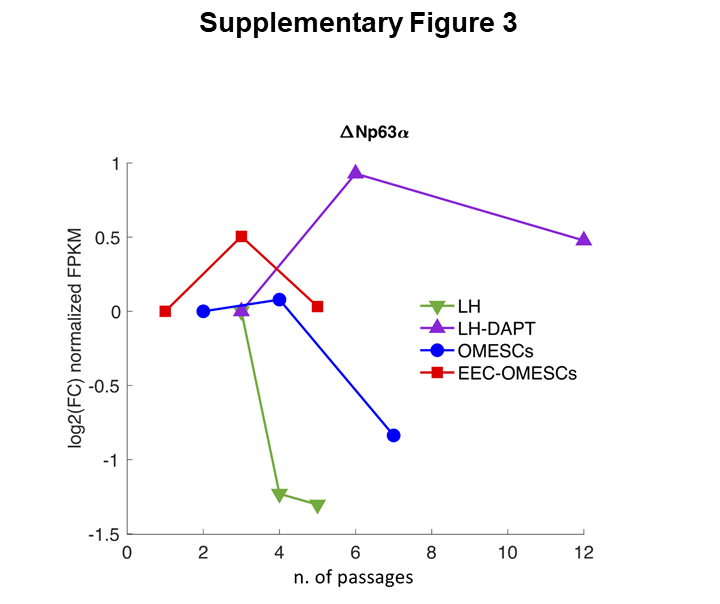

Supplement: Supplementary file 3 — Figure S3 [file JCMM-26-3977-s008.tiff]

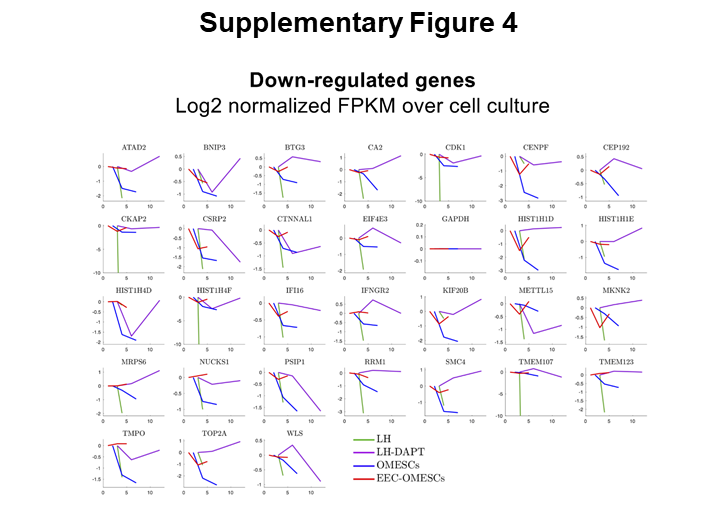

Supplement: Supplementary file 4 — Figure S4 [file JCMM-26-3977-s007.tiff]

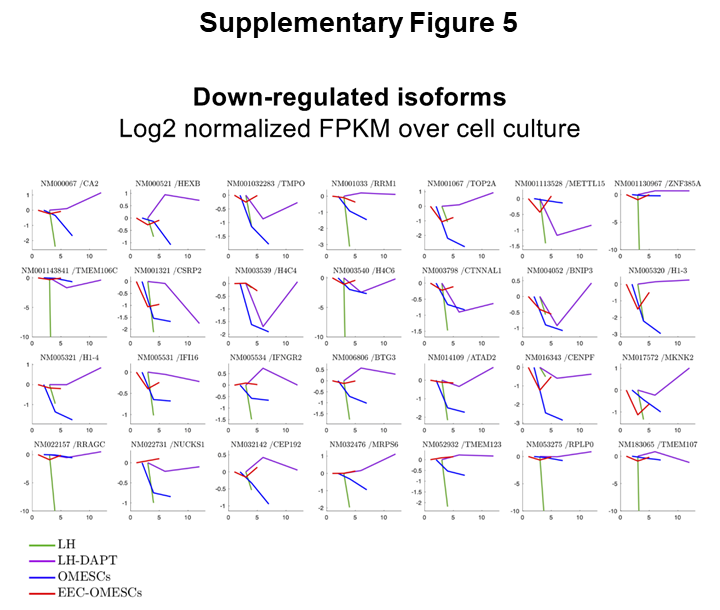

Supplement: Supplementary file 5 — Figure S5 [file JCMM-26-3977-s001.tiff]

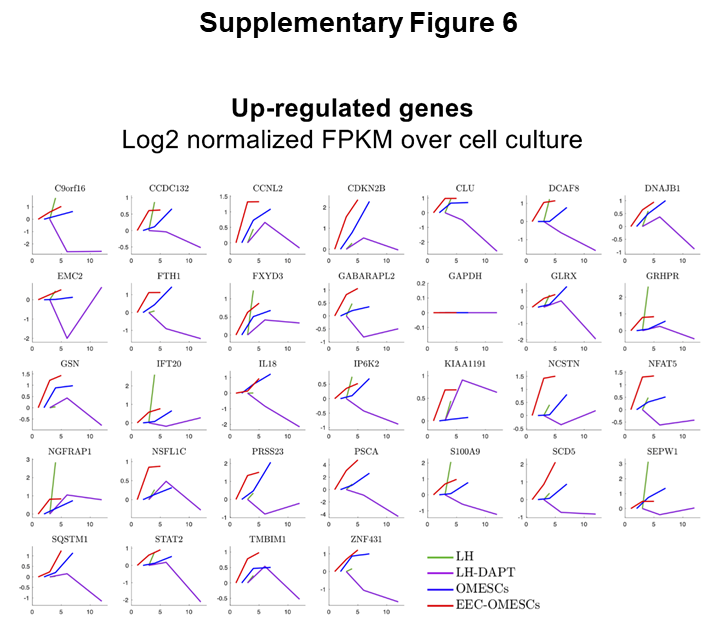

Supplement: Supplementary file 6 — Figure S6 [file JCMM-26-3977-s003.tiff]

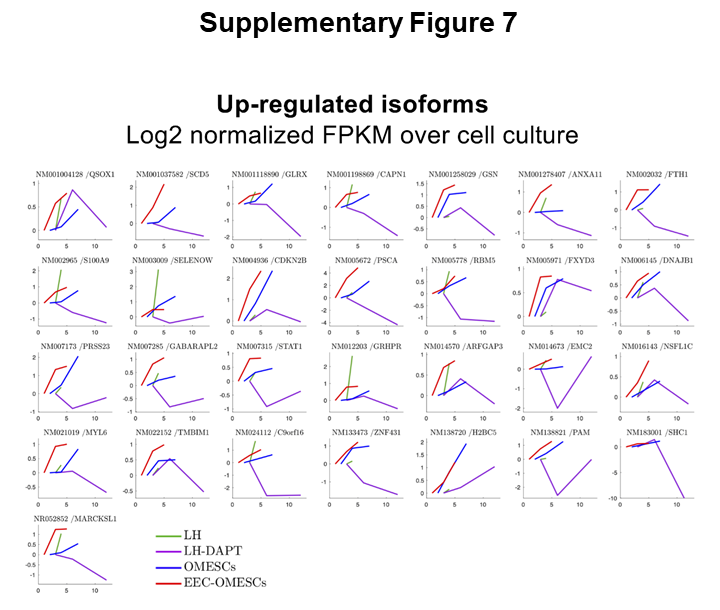

Supplement: Supplementary file 7 — Figure S7 [file JCMM-26-3977-s009.tiff]
