# Supplementary material for: Analysis and pharmacological modulation of senescence in human epithelial stem cells
Source: J Cell Mol Med. 2022 Jun 15;26(14):3977–94. doi: 10.1111/jcmm.17434 (PMC9279594; doi:10.1111/jcmm.17434)
Supplement: Supplementary file 8 — Table S1 [file JCMM-26-3977-s002.docx]

| **Upregulated genes:** | **Type** | **Description** | **Function** |
| --- | --- | --- | --- |
| C9orf16 | / | Chromosome 9 Open Reading Frame 16 | Uncharacterized protein |
| CCDC132 | / | Coiled-Coil Domain Containing 132 | Endosome-associated recycling protein (EARP) complex involved in endocytic recycling |
| CCNL2 | Cyclin family | Cyclin L2 | Involved in pre-mRNA splicing. CCNL2 encodes a cyclin that induces cell cycle arrest, promotes apoptosis, and its overexpression has been shown to inhibit growth in several types of cancer (Zhuo et al. 2009) |
| CDKN2B | Cyclin-dependent kinase inhibitor family | Cyclin-dependent kinase 4 inhibitor B | Cell growth regulator that controls cell cycle G1 progression |
| CLU | Molecular chaperone | Clusterin | Responsible for aiding protein folding of secreted proteins, and implicated in pro- or antiapoptotic processes. Overexpression of human clusterin increased stress resistance and significantly extended lifespan.. |
| DCAF8 | WD repeat-containing protein (protein ubiquitination) | DDB1 And CUL4 Associated Factor 8 | May function as a substrate receptor for CUL4-DDB1 E3 ubiquitin-protein ligase complex. |
| DNAJB1 | Molecular chaperone | DnaJ Heat Shock Protein Family (Hsp40) Member B1 | Stimulates the ATPase activity of Hsp70 heat-shock proteins in order to promote protein folding and prevent misfolded protein aggregation. |
| EMC2 | Endoplasmic reticulum membrane protein | ER membrane protein complex subunit 2 | Component of the ER membrane protein complex (EMC), which is composed of EMC1, EMC2, EMC3, EMC4, EMC5 and EMC6. |
| FTH1 | Heavy subunit of ferritin  (iron storage) | Ferritin Heavy Chain 1 | Storage of iron in a soluble and nontoxic state. Iron homeostasis plays an important role in a wide range of cellular activities, including proliferation, cell cycle, programmed cell death, and development |
| FXYD3 | Small membrane protein | FXYD domain-containing ion transport regulator 3 | Cell surface regulator modulating the function of ion pumps and ion channels. |
| GABARAPL2 | Ubiquitin-like modifier | Gamma-aminobutyric acid (GABA) A receptor-associated protein-like 2 | Involved in intra-Golgi traffic and autophagy. Plays a role in mitophagy which contributes to regulate mitochondrial quantity and quality by eliminating the mitochondria to a basal level to fulfill cellular energy requirements and preventing excess ROS production. |
| GLRX | Metabolic enzyme (glutaredoxin family) | Glutaredoxin-1 | Cytoplasmic enzyme catalyzing the reversible reduction of glutathione-protein mixed disulfides. This enzyme highly contributes to the antioxidant defense system. |
| GRHPR | Metabolic enzyme | Glyoxylate reductase/hydroxypyruvate reductase | Enzyme with hydroxypyruvate reductase, glyoxylate reductase, and D-glycerate dehydrogenase enzymatic activities. |
| GSN | Actin-binding protein | Gelsolin | Key regulator of actin filament assembly and disassembly |
| IFT20 | Transport protein | Intraflagellar Transport 20 | Involved in trafficking of proteins from the Golgi body, including recycling of immune signalling components. |
| IL18 | Cytokine (Interleukin-1 family) | Interleukin-18 (interferon-gamma inducing factor) | Potent immunomodulatory cytokine which promotes T‐helper (Th) 1 and cytotoxic responses. |
| IP6K2 | Protein kinase  (inositol phosphokinase family) | Inositol Hexakisphosphate Kinase 2 | Involved in growth suppressive and apoptotic activities |
| KIAA1191 | Oxidoreductase activity | Putative monooxygenase p33MONOX | Potential NADPH-dependent oxidoreductase. May be involved in the regulation of neuronal survival, differentiation and axonal outgrowth. |
| NCSTN | Transmembrane glycoprotein (integral component of the multimeric gamma-secretase complex) | Nicastrin | Essential subunit of the gamma-secretase complex, an endoprotease complex that catalyzes the intramembrane cleavage of integral membrane proteins such as Notch receptors and APP (amyloid-beta precursor protein).The gamma-secretase complex plays a role in Notch and Wnt signaling cascades and regulation of downstream processes via its role in processing key regulatory proteins, and by regulating cytosolic CTNNB1 levels. |
| NFAT5 | Transcription factor | Nuclear factor of activated T-cells 5 | Crucial component of the osmotic-stress response pathway. |
| NGFRAP1 | NGF associated protein | Nerve growth factor receptor-associated protein 1 Alternative name BEX3: Brain-Expressed X-Linked Protein 3 | May be a signaling adapter molecule involved in p75NTR-mediated apoptosis induced by NGF. Plays a role in zinc-triggered neuronal death (By similarity). May play an important role in the pathogenesis of neurogenetic diseases. |
| NSFL1C | SEP domani superfamily | NSFL1 cofactor p47 | Golgi organization and biogenesis and membrane fusion |
| PRSS23 | Trypsin family of serine proteases | Serine protease 23 | Serine-type endopeptidase activity |
| PSCA | Membrane glycoprotein | Prostate stem cell antigen | May be involved in the regulation of cell proliferation. Has a cell-proliferation inhibition activity in vitro. |
| S100A9 | Calcium- and zinc-binding protein | S100 Calcium Binding Protein A9 | Involved in the regulation of a number of cellular processes such as cell cycle progression and differentiation. |
| SCD5 | Endoplasmic reticulum membrane | Stearoyl-CoA Desaturase 5 | Key regulator of energy metabolism. Significantly expressed in primary melanoma, but becomes barely detectable at tumor advanced stages. |
| SEPW1 | Redox enzyme | Selenoprotein W, 1 | Involved in redox-related processes, muscle growth and differentiation, and in the protection of neurons from oxidative stress during neuronal development. |
| SQSTM1 | Autophagy receptor | Sequestosome 1 | Functions as a bridge between polyubiquitinated cargo and autophagosomes. |
| STAT2 | Transcription activator | Signal transducer and activator of transcription 2 | STAT2 forms an integral part of the signaling machinery that links ligation of innate interferon receptors to the protective changes in gene transcription. |
| TMBIM1 | Death receptor binding | Transmembrane BAX inhibitor motif-containing protein 1 | Located in endosomal/lysosomal membranes and Golgi apparatus. Inhibitory activities in different setting of apoptosis. |
| ZNF431 | Krueppel C2H2-type zinc-finger family | Zinc Finger Protein 431 | Sequence-specific DNA binding transcriptional repressor. |
| **Upregulated isoforms:** | **Type** | **Description** | **Function** |
| QSOX1 (NM001004128) | Enzyme | Sulfhydryl oxidase 1 | QSOX1 participates in the folding and stability of proteins and thus could regulate the biological activity of its substrates in the secretory pathway and/or outside the cell. Protective role of QSOX1 against apoptosis. |
| CAPN1  (NM001198869) | Enzyme (Proteinase) | Calpain 1 | Calpain activation has been implicated in various aging phenomena and diseases of late life, including cataract formation, erythrocyte senescence, diabetes mellitus type 2, hypertension, arthritis, and neurodegenerative disorders. |
| ANXA11 (NM001278407) | Phospholipid-binding protein | Annexin A11 | Required for midbody formation and completion of the terminal phase of cytokinesis. |
| RBM5  (NM005778) | Spliceosome component | RNA Binding Motif Protein 5 | Nuclear RNA binding protein that is a component of the spliceosome A complex. The encoded protein plays a role in the induction of cell cycle arrest and apoptosis through pre-mRNA splicing of multiple target genes including the tumor suppressor protein p53. |
| STAT1  (NM007315) | Transcription factor | Signal transducer and activator of transcription 1 | Key role in many gene expressions that cause survival of the cell, viability or pathogen response. |
| ARFGAP3  (NM014570) | GTPase-activating protein | ADP-ribosylation factor GTPase-activating protein 3 | Associates with the Golgi apparatus and regulates the vesicular trafficking pathway. |
| MYL6  (NM021019) | Cellular motor protein | Myosin Light Chain 6 | Motor proteins that share the common features of ATP hydrolysis (ATPase enzyme activity), actin binding and potential for kinetic energy transduction. |
| H2BC5  (NM138720) | Members of the H2 class of histones | H2B Clustered Histone 5 | Core component of nucleosome |
| PAM  (NM138821) | Enzyme | Peptidylglycine Alpha-Amidating Monooxygenase | Involved in the biosynthesis of many signaling peptides and some fatty acid amides. |
| SHC1  (NM183001) | Adaptor proteins | SHC-transforming protein 1 | It appears to play a role in the regulation of intra-cellular redox levels, signal transduction, and apoptosis |
| MARCKSL1 (NR052852) | F-actin binding | MARCKS-related protein (Myristoylated Alanine Rich Protein Kinase C Substrate like 1) | Role in cytoskeletal regulation, protein kinase C signaling and calmodulin signaling. The encoded protein affects the formation of adherens junction. |

**Supplementary Table 1:** List of upregulated genes and isoforms.
