# Supplementary material for: Analysis and pharmacological modulation of senescence in human epithelial stem cells
Source: J Cell Mol Med. 2022 Jun 15;26(14):3977–94. doi: 10.1111/jcmm.17434 (PMC9279594; doi:10.1111/jcmm.17434)
Supplement: Supplementary file 9 — Table S2 [file JCMM-26-3977-s005.docx]

| **Downregulated genes:** | **Type** | **Description** | **Function** |
| --- | --- | --- | --- |
| ATAD2 | Histone chaperone | ATPase family AAA domain containing 2 | Implicated in nucleosome density regulation by histone H3-H4 loading or removal. |
| BNIP3 | Mitochondrial protein | BCL2 interacting protein 3 | A bifunctional mitochondrial proteins: can induce cell death (apoptosis, or necrosis following mitochondrial membrane permeabilization), autophagy and/or mitophagy through their LIR domains. |
| BTG3 | Anti-proliferative BTG/Tob protein family | BTG anti-proliferation factor | Member of the BTG/Tob family. This family has structurally related proteins that appear to have antiproliferative properties. This encoded protein might play a role in neurogenesis in the central nervous system. |
| CA2 | Zinc metalloenzymes | Carbonic anhydrase 2 | Catalyzes reversible hydration of carbon dioxide. Defects in this enzyme are associated with osteopetrosis and renal tubular acidosis. |
| CDK1 | Serine/threonine kinase | Cyclin-dependent kinase 1 | Regulator of the cell cycle |
| CENPF | Centromere protein | Centromere protein F | Kinetochore function and chromosome segregation in mitosis. |
| CEP192 | Centrosomal protein | Centrosomal Protein 192 | Mitotic centrosome maturation and bipolar spindle assembly |
| CKAP2 | Cytoskeletal protein | Cytoskeleton associated protein 2 | Stabilizes microtubules and plays a role in the regulation of cell division. |
| CSRP2 | LIM domain protein | Cysteine and glycine rich protein 2 | Involved in regulatory processes important for development and cellular differentiation. |
| CTNNAL1 | Alpha catenin family | Catenin Alpha Like 1 | Involved in cell adhesion. |
| EIF4E3 | Translational initiation factors | Eukaryotic Translation Initiation Factor 4E Member 3 | RNA binding and translation initiation factor activity in somatic cells modulates ageing in *Caenorhabditis elegans* |
| HIST1H1D | Histones | H1 Histone family, member D | Basic proteins that associate with DNA in the nucleus and have an important role in establishing and maintaining higher order chromatin structures. |
| HIST1H1E |  | H1 Histone family, member E |  |
| HIST1H4D |  | H4 Histone family, member D |  |
| HIST1H4F |  | H4 Histone family, member F |  |
| IFI16 | Cytokines | Interferon-gamma-inducible protein 16 | Modulates proliferation, survival and differentiation of different cell lineages. |
| IFNGR2 | Non-ligand-binding beta chain of the gamma interferon receptor. | Interferon Gamma Receptor 2 | Involved in cytokine receptor activity and interferon-gamma receptor activity |
| KIF20B | Kinesins | Kinesin Family Member 20B | Required for completion of cytokinesis |
| METTL15 | Methyltransferase activity and rRNA(cytosine-N4-)-methyltransferase activity. | Methyltransferase Like 15 | Mitoribosome small subunit biogenesis and mitochondrial translation. |
| MKNK2 | Protein kinase | MAPK Interacting Serine/Threonine Kinase 2 | Role in the response to environmental stress and cytokines. |
| MRPS6 | Mitochondrial protein | Mitochondrial Ribosomal Protein S6 | Linked to aging and longevity in model organisms (i.e., mice, *Caenorhabditis elegans*). |
| NUCKS1 | Chromosomal, vertebrate-specific protein | Nuclear Casein Kinase And Cyclin Dependent Kinase Substrate 1 | Chromatin remodeling and regulates cellular events such as replication, transcription, and chromatin condensation |
| PSIP1 | Transcriptional coactivator | PC4 and SFRS1 interacting protein 1 | Involved in neuroepithelial stem cell differentiation and neurogenesis. |
| RRM1 | Enzyme | Ribonucleotide Reductase Catalytic Subunit M1 | Provides the precursors necessary for DNA synthesis. Conversion of ribonucleotides into deoxyribonucleotides |
| SMC4 | Subunit of the 13S condensin complex | Structural maintenance of chromosomes protein 4 | Involved in chromosome condensation |
| TMEM107 | Transmembrane protein | Transmembrane Protein 107 | Regulates ciliogenesis and ciliary protein composition. |
| TMEM123 | Transmembrane protein | T  Transmembrane Protein 123 | Implicated in oncotic cell death, characterized by cell swelling, organelle swelling, vacuolization and increased membrane permeability. |
| TMPO | Inner nuclear membrane protein | Thymopoietin | Involved in the structural organization of the nucleus and in the post-mitotic nuclear assembly. |
| TOP2A | Nuclear enzyme | DNA Topoisomerase II Alpha | Involved in processes such as chromosome condensation, chromatid separation, and the relief of torsional stress that occurs during DNA transcription and replication |
| WLS | Transmembrane protein | Wnt Ligand Secretion Mediator | Necessary for Wnt protein secretion and stem cell population maintenance |

| **Downregulated isoforms:** | **Type** | **Description** | **Function** |
| --- | --- | --- | --- |
| HEXB  (NM000521) | Hexosaminidase (glycosylhydrolase) | Hexosaminidase subunit beta (HEXB), transcript variant 1 | Responsible for turnover of the glycosphingolipid ganglioside GM2 |
| ZNF385A (NM001130967) | Zinc finger RNA-binding protein | Zinc finger protein 385A (ZNF385A), transcript variant 1 | Regulatory proteins that act as transcription factors, bind single- or double-stranded RNA, or interact with other proteins |
| TMEM106C (NM001143841) | Transmembrane protein | Transmembrane protein 106C (TMEM106C), transcript variant 4 | Endoplasmic reticulum membrane protein overexpressed in cancer |
| H4C6  (NM003540) | Histones | H4 clustered histone 6 (H4C6) | Core component of nucleosome |
| H1-3  (NM005320) |  | H1.3 linker histone, cluster member (H1-3) |  |
| H1-4 (NM005321) |  | H1.4 linker histone, cluster member (H1-4) |  |
| RRAGC  (NM022157) | Monomeric guanine nucleotide-binding protein | Ras related GTP binding C (RRAGC), transcript variant 1 | Plays a crucial role in the cellular response to amino acid availability through regulation of the mTORC1 signaling cascade |
| RPLP0  (NM053275) | Ribosomes protein | Ribosomal protein lateral stalk subunit P0 (RPLP0), transcript variant 2 | Component of the 60S subunit |

**Supplementary Table 2:** List of dowregulated genes and isoforms.
